# Joining of the Laminated Electrical Steels in Motor Manufacturing: A Review

**DOI:** 10.3390/ma13204583

**Published:** 2020-10-15

**Authors:** Cunjuan Xia, Hongze Wang, Yi Wu, Haowei Wang

**Affiliations:** State Key Laboratory of Metal Matrix Composites, School of Materials Science & Engineering, Shanghai Jiao Tong University, No. 800 Dongchuan Road, Shanghai 200240, China; xiacunjuan@sjtu.edu.cn (C.X.); eagle51@sjtu.edu.cn (Y.W.); hwwang@sjtu.edu.cn (H.W.)

**Keywords:** electrical steel, joining and welding, microstructure, magnetic property, mechanical property

## Abstract

In recent years, the motor has been increasingly used to replace the conventional gasoline engine for carbon emission reduction, and the high-performance motor is urgently required. The stator and rotor in a motor are made of hundreds of joined and laminated electrical steels. This paper covers the current research in joining the laminated electrical steels for the motor application, together with the critical assessment of our understanding. It includes the representative joining method, modeling of the joining process, microstructure of the weld zone, mechanical strength and magnetic properties. The gaps in the scientific understanding, and the research needs for the expansion of joining laminated electrical steels, are provided.

## 1. Introduction

As a machine to transform the electrical energy into mechanical energy, a motor has been widely used as the traction machine in industry equipment [1,2,3,4,5,6,7], e.g., electrical vehicle, electrical airplane, electric ship, and so on. Electrical steel [8,9,10,11], a high silicon (2–5.5 wt% Si) [12,13] and thin sheet (0.2–0.65 mm) steel [14], is the soft magnetic material for the stator and rotor in a motor [15,16,17]. The addition of silicon to iron results in a decrease in coercivity and an increase in resistivity [12,18,19,20,21,22]. Furthermore, the reduction of the sheet thickness results in the reduction of the eddy current loss in the electrical steel when put in the alternating magnetic field environment [14,23,24]. The stator and rotor in a motor are made of hundreds of laminated and joined thin electrical steel sheets [25], which could reduce the eddy current loss and improve efficiency. There are insulation coatings on both sides of the electrical steel sheet to cut off the interlaminar eddy current when hundreds of electrical steels are laminated in the motor application [26,27,28,29,30,31,32,33,34,35,36]. Generally, the goal of joining the laminated electrical steels is to ensure the mechanical strength of the laminations [37], while the joining process will lead to the degradation of the magnetic properties due to the damage of the insulation coating [38], the modification of the microstructure [39,40], the introduction of the residual stress [41], and so on. It is a great challenge to reach the trade-off between mechanical strength and magnetic properties [42]. Besides, the structure of the laminated electrical steels is different from the conventional lapped or butted sample, and the conventional knowledge about joining may not work for joining the laminations. Finally, it is important to study the joining of the laminated electrical steels, which could speed up the roadmap towards high-quality motor manufacturing.

**Figure 1:**
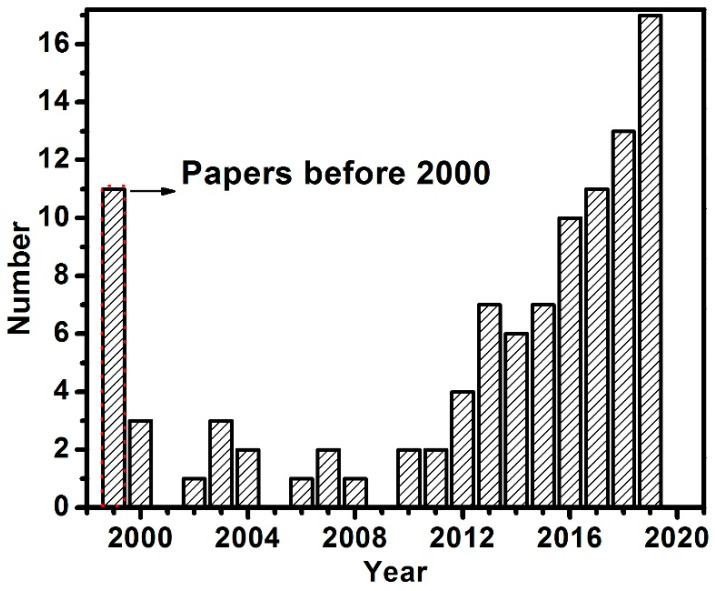
The number of papers in Scopus varying with the year when searching with the combined keywords of “electrical steel” and “welding” or “joining” in the title, abstract and keywords.

Figure 1 shows the number of papers in the Scopus database about the joining and welding of laminated electrical steels. As shown, it is an emerging research topic with rapidly increasing speed in the last decade. There is no doubt that more research about this topic will appear along with the rapid increase of the electric vehicle market. In this manuscript, the current progress in joining the laminated electrical steels is summarized, the gaps in the scientific understanding and the research needs in this field are provided based on the authors’ research experiences.

## 2. The Representative Joining Method

**Figure 2:**
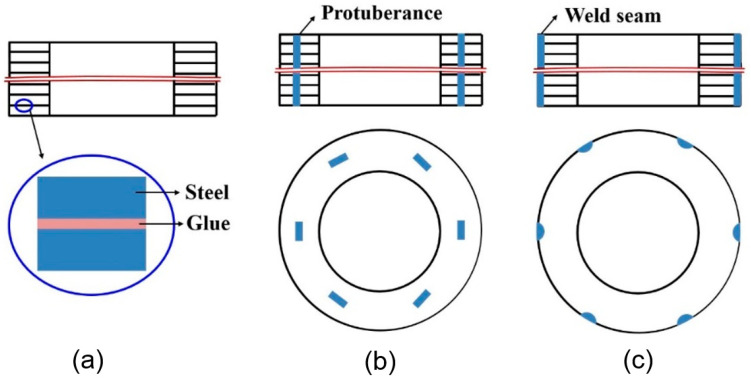
Schematic of the representative join methods for laminated electrical steels: (**a**) Glue join; (**b**) mechanical join; (**c**) fusion welding [40].

Currently, the joining method for the laminated electrical steels could be generally categorized into three types: glue join [26,43], mechanical join [44] and fusion welding [45], as shown in Figure 2 [40]. The advantage of the glue-join method was that it did not destroy the insulation coating. Kaido et al. [26] measured the magnetic and mechanical characteristics of adhesive coating non-oriented electrical steel sheet cores in the conditions of motor and found that the deteriorations of iron losses and exciting currents by adhesion were less than those by welding. Schoppa et al. [46] coated the electrical steel laminations with the adhesive varnish, then the laminations were stuck together during a thermally activated process. Their experimental results showed that the increase of the specific core loss after sticking was very low, and they concluded that sticking was from the magnetic point of view one of the best methods of assembling laminations into magnetic cores. The glue-join method also allowed homogenous electrical isolation, reduced acoustic emission, and behaved high thermal conductivity in service [26,43]. Generally, the composition of the glue varies with the supplier, including the organic glue, inorganic glue and their combinations. However, the biggest obstacle for the large-scale application of this technique was the concern about the mechanical failure of the adhesion under the periodic load condition at an elevated temperature during the operation of the motor [47]. Besides, the cost was also higher than the other joining methods [46].

Both mechanical joining [44,48,49,50] and fusion welding [37,38,46,51,52] are widely used to join the laminated electrical steels at present. Senda et al. [44] compared the effects of two representative V-type mechanical interlocking methods, dowel formation and dowel jointing on the magnetic properties of the joined ring core sample made of the electrical steel laminations, they found that two methods showed comparable contributions to iron loss increase at low frequencies (e.g., 50 Hz), whereas, increases in iron loss due to dowel jointing were greater than those due to dowel formation at high frequencies. Imamori et al. [49] investigated the influence of interlocking on the magnetic properties of ring cores by measurement, and they observed that the inverse of permeability and iron loss increased linearly with the number of interlocks. The mechanical joining process is usually combined with the punching process in the progressive stamping die process. Finally, the cost of the mechanical joining process is a bit lower than that of the welding process. The disadvantage of the mechanical joining method is the lower strength at the direction perpendicular to the electrical steel surface compared to that of the fusion welded joint. Besides, the mechanically joined joint has a lower fatigue life under the periodic loads than that of the welded joint. In the case with the high strength requirement, several fusion welding passes were jointly used to enhance the strength of the mechanically joined sample.

**Figure 3:**
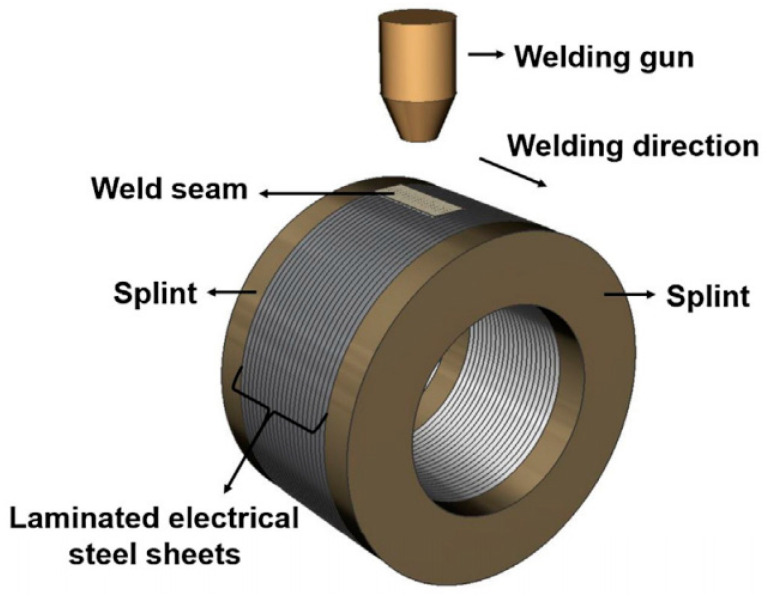
Schematic of the fusion welding process [40].

The heat source used in fusion welding of the electrical steel laminations includes laser [37], electron beams [53], plasma arcs [39], electric arcs (TIG, GTA, CMT) [51,54], and so on. As a high efficiency and high-quality fusion welding method, laser welding was thought to be a potential method for welding of the electrical steel laminations in the high-performance motor application [37,40,42,51,55]. Compared to the other fusion welding methods, laser welding could achieve a smaller heat affected zone, induce lower residual stress, and finally obtain the welded electrical steel laminations with higher magnetic properties. Figure 3 shows the schematic of laser welding of laminated electrical steel laminations [40]. The moving energy beam melt the edge of the laminations continuously and the effective joint was formed at the interfaces of the laminations.

Table 1 shows the representative research in the joining of laminated electrical steels. The critical factors affecting joining the laminated electrical steel laminations are as follows: (a) the special structure of the laminations made of hundreds of electrical steel sheets; (b) the insulation coating on both sides of the electrical steel sheet, which affects the dynamics of the molten pool during the fusion welding process because of the entrapped bubbles due to the pyrolysis of the coating and may induce pores in the weld seam; (c) the comprehensive requirement of the strength and magnetic property. The following sections will summarize the current research in joining of laminated electrical steels, which provides a better understanding of the joining process with great demands from the industry.

## 3. Characteristics of the Joined Zone

**Figure 4:**
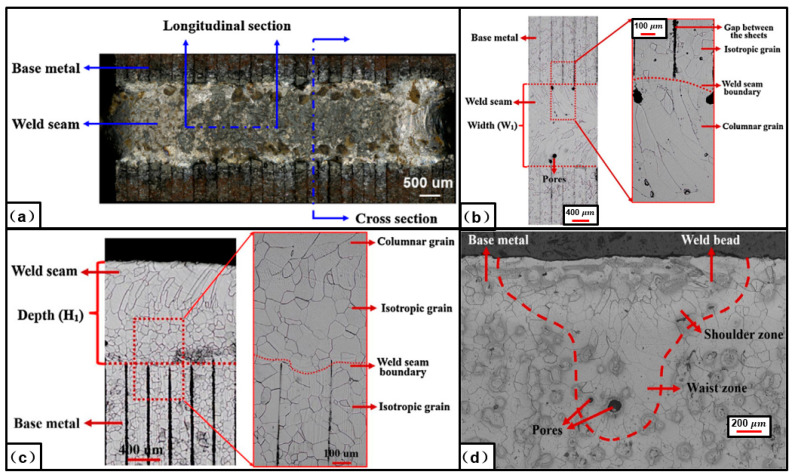
Characteristics of the weld seam in laser-welded laminated electrical steels made with a welding speed of 10 mm/s: (**a**) Overall view; (**b**) upper surface; (**c**) longitudinal section; (**d**) cross section [40].

**Figure 5:**
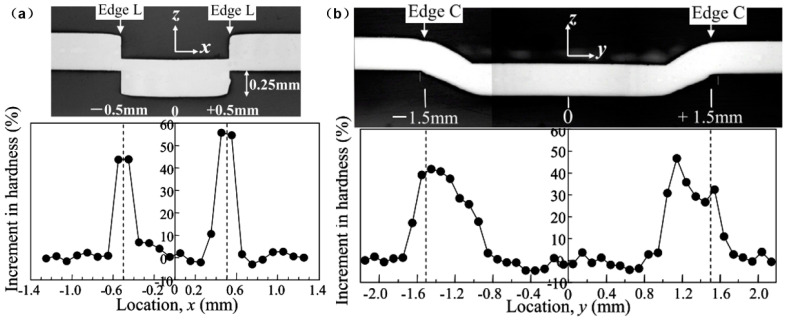
Cross-sectional view and hardness distribution of the mechanical interlock joint: (**a**) Along the short edge direction; (**b**) along the long edge direction [44]. The microhardness was measured every 0.1 mm.

As high silicon (2–5.5 wt% Si) [12,13] and low carbon steel, the basic ferric phase appears in the base material of the electrical steel. Wang et al. [40] investigated the surface morphology and microstructure of the laser-welded electrical steel laminations joint. As shown in Figure 4, the surface of the weld seam has a good quality and there is no obvious defect there. Because of the high content of the silicon element, the weld seam zone is still made of the ferric phase even if it solidifies with a high cooling rate. Epitaxial growth based on the grains at the base metal happens, and columnar grains growing towards the direction of the temperature gradient appears in the weld seam zone. Small pore defects are observed in the zone near the boundary of the weld seam [40]. This research provides an insight into the microstructures at both the surface and the interior of the laser welded electrical steel joint. Senda et al. [44] investigated the characteristics and hardness distribution of the mechanically interlocked joint. As shown in Figure 5, the large local deformation at the edge of the dowel led to the interlock between the laminations, and hardness of the edge zone increased due to the strengthening effect. This research provides a clear insight into the shape and hardness of the mechanical interlocked joint. However, current research about the characteristics of the joined zone of the electrical steel laminations is still limited, much work is required to clarify the processing parameter window for defect-free joint, the grain size and orientation, and so on.

## 4. Simulation of the Joining Process

**Figure 6:**
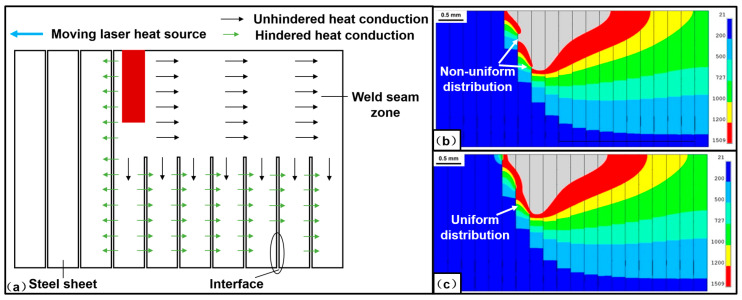
(**a**) Schematic of the heat transfer process at the longitudinal section of the weld seam during the welding process; (**b**) simulated non-uniform temperature distribution at the longitudinal section of the weld seam because of the hindrance of the interface on heat transfer; (**c**) simulated uniform temperature distribution at the longitudinal section of the weld seam when the interface was melted due to absorbing the energy of laser [47].

The simulation will act as a useful tool to reveal the mechanism underlying the process of joining the laminated electrical steels. Though this method has been widely used in the field of joining and welding of various materials [58,59], the research about the simulation of joining the laminated electrical steels is still at the initial stage [47]. One of the difficulties in the simulation of welding the laminated electrical steels is how to describe the effect of the interfaces on the heat transfer during the welding process. Wang et al. [47] developed a thermal analysis finite element model in ANSYS to calculate the temperature distribution and analyze the evolution of the interfaces during laser welding of the laminated electrical steel laminations, as shown in Figure 6. In the model, the technique of “birth” and “death” element was used to describe the effects of the interfaces on the heat transfer, where two groups of values for the thermal contact conductance were used respectively to describe the heat transfer ability of the interface before and after melting. Based on this model, the simulated molten pool fit well with the experimental one. In the future, much work should be done to analyze the flows of the material at the interface, the evolution of the temperature and residual stress, and so on.

## 5. Mechanical Properties

**Figure 7:**
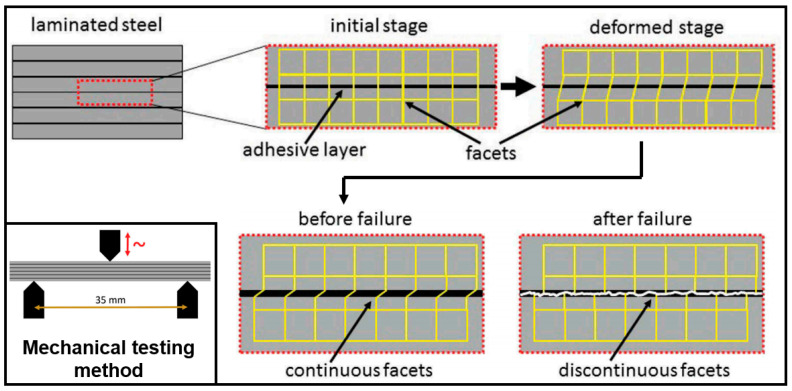
Schematic illustration of surface discretization by facets required for full-field strain analysis at an initial stage and an idealized deformed stage, before (**left**) and after (**right**) ultimate failure, the schematic illustration of dynamic mechanical analysis setup in the three-point bending mode with relevant support distance was also laid out at the left corner [26]. In this literature, the adhesive was first applied to square steel sheets using a doctor knife and was pre-crosslinked to a non-sticky stage, then the steel sheets with pre-cured adhesive were stacked to form a 6-layer laminate and cured at 160 °C for 90 min.

The greatest challenge in joining the laminated electrical steels is to achieve excellent mechanical properties and magnetic properties at the same time. Though the stator and rotor in a motor do not have high requirements for the joint strength between the laminations, it is still important to evaluate the shear strength and the fracture shear strain of the joint. Figure 7 shows the schematics for measuring the shear strength of the bonded laminations and the fracture strain. In the sample preparation stage, the adhesive was first applied to square steel sheets using a doctor knife and was pre-crosslinked to a non-sticky stage, then the steel sheets with pre-cured adhesive were stacked to form a 6-layer laminate and cured at 160 °C for 90 min [26]. The three-point bending experiment was used to measure the shear strength of the bonded laminations, and the shear fracture strain could be measured by the digital image correlation method [26]. This is a useful method to evaluate the mechanical properties of the bonded laminations, while may not work well for the mechanically interlocked or fusion-welded ones due to the small joining area in these two methods.

**Figure 8:**
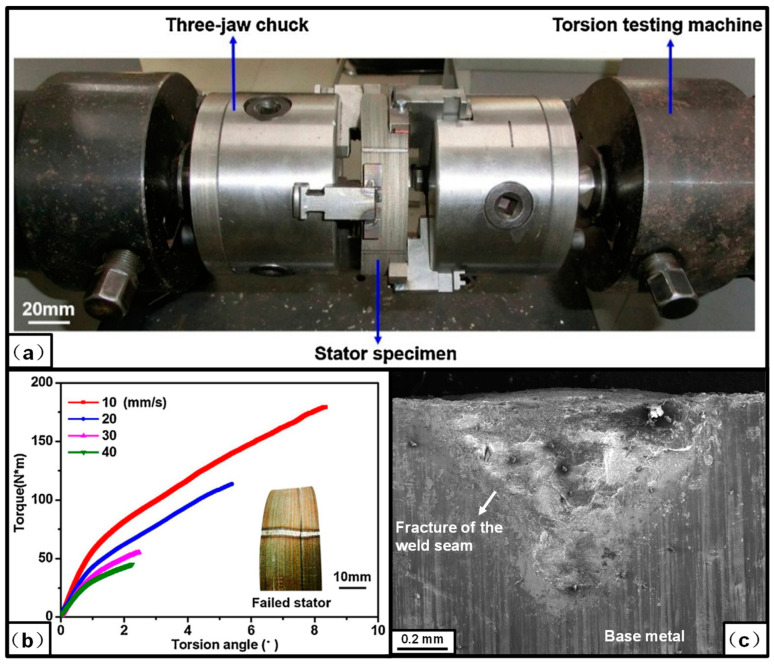
(**a**) Experimental system to measure the torsion properties of the fusion-welded laminations; (**b**) experimental torsion properties of the laser-welded laminations; (**c**) characteristics of the fracture (this subfigure is reedited based on the original image). The fracture appeared at the weld zone along with the interface of the welded laminations and the fracture mode belonged to the interfacial fracture, characteristics of the fracture showed that brittle fracture happened at the interface on the torsion load condition [40].

**Figure 9:**
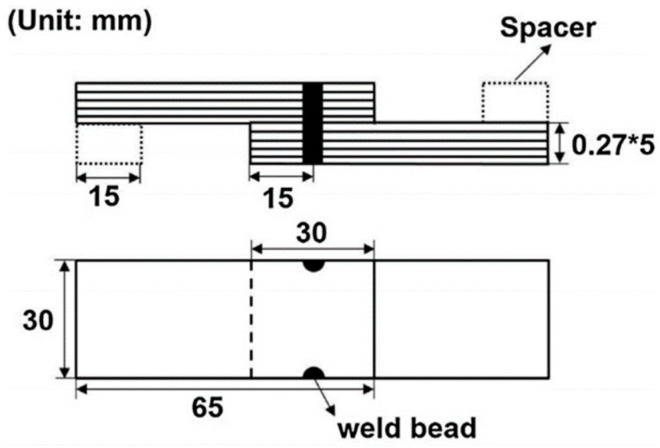
Schematic of the specimen to evaluate the lap strength of the fusion-welded laminated electrical steels [51]. Weld seams were set symmetrically at two sides of the specimen to keep the balance during the lap shear process.

To measure the torsion strength of the fusion-welded ring electrical steel laminations, Wang et al. [40] developed a three-jaw chuck adaptor, which was assembled with a torsion testing machine, as shown in Figure 8. This system was successfully used to measure the torsion-property of the laser welded ring sample, and could also be expanded to measure the torsion property of the ring sample joined by mechanical interlock and glue, as well as the joined stator and rotor in an actual motor. In the actual application, the rectangular electrical steel laminations sample has been widely used at the stage of hunting the processing parameter window. To measure the shear strength of the weld seam in the rectangular sample, Zhang et al. [51] designed a special structure, where two weld seams were symmetrically distributed at each edge of the sample, as shown in Figure 9. These systems act as useful tools to evaluate the strength of the joined laminations with different geometric shapes. In the future, the digital image correlation method could also be used to measure the local strain during the loading process.

## 6. System to Measure the Magnetic Properties

**Figure 10:**
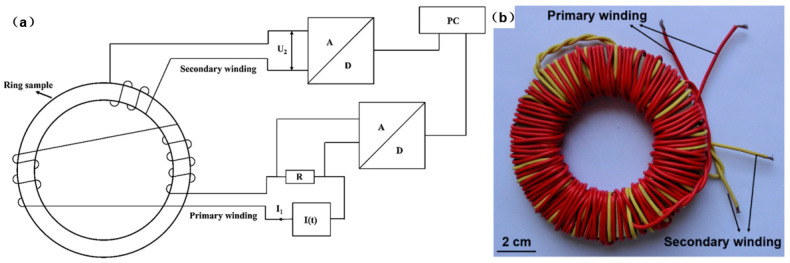
System to measure the magnetic properties: (**a**) The schematic; (**b**) the winded sample [40]. I_1_ was the current in the primary winding, I(t) represented the module generating the current varying with the time, R represented the resistance module, U_2_ was the voltage between the secondary winding, A represented the analog signal processing module, D represented the digital signal processing module, and PC represented the personal computer.

Magnetic properties are the other indicators to evaluate the performance of the welded electrical steel laminations except for the torsion strength. Wang et al. [40] adopted an experimental system to measure the magnetic properties of the welded electrical steel laminations, as shown in Figure 10. The measurement principle of the system was as follows [40]:

(1) The iron loss of the welded laminations could be calculated by Equation (1):

(1) $$P_{s}=\frac{1}{\rho_{1}T}\int_{t}H\frac{dB}{dt}dt$$

where $\rho_{1}$ was the density of the electrical steel sample, *T* was the period of time in the measurement.

(2) The magnetic field H was calculated by Equation (2):

(2) $$H=\frac{N_{1}I_{1}}{L}$$

where *N*_1_ was the primary winding turns, *I*_1_ was the current in the primary winding, and *L* was the length of the equivalent magnetic circuit, which could be calculated by Equation (3):

(3) $$L=\pi \left(D_{1}+D_{2}\right)/2$$

where *D*_1_ was the external diameter of the ring laminations, and *D*_2_ was the internal diameter of the ring laminations.

(3) the magnetic flux density through the laminations was calculated by Equation (4):

(4) $$B=-\frac{1}{N_{2}S}\int U_{2}dt$$

where *N*_2_ was the secondary winding turns, *U*_2_ was the voltage between the secondary winding, and S was the section area of the secondary winding, which could be calculated by Equation (5):

(5) $$S=h\left(D_{1}-D_{2}\right)/2$$

where *h* was the height of the ring laminations.

Before the experiment, the welded sample was winded, and the numbers of the primary winding turns and the secondary winding turns were counted, respectively. The other papers also mentioned similar experimental systems to measure the magnetic properties of the electrical steel laminations [48,60,61], and all these systems were developed based on the same principle.

## 7. Eddy Current Loss Increase Induced by the Joining Process

**Figure 11:**
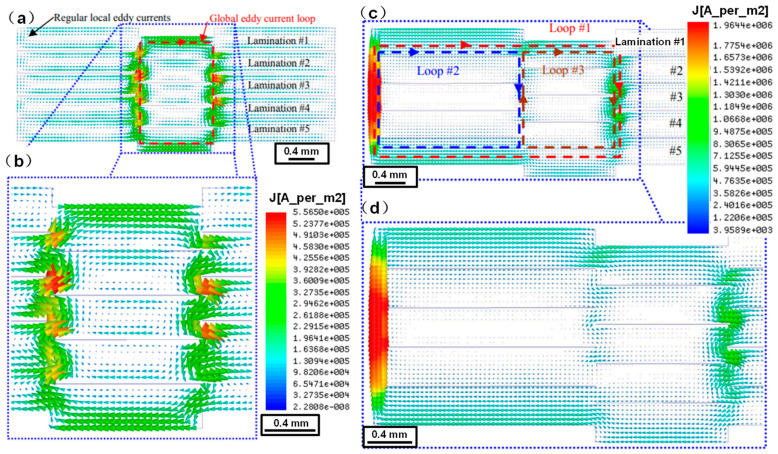
Cross-section view of: (**a**,**b**) interlocked electrical steel laminations; (**c**,**d**) with nickel coating at the edge. (**a**,**c**) entire cross section. (**b**) interlock area detail of (**a**); (**d**) interlocking/nickel coating area detail of (**c**) [62]. The arrows represent the eddy current density and direction, five laminations, 400 Hz/1.0 T. The length scales have been added to the figure and the font size of the eddy current scales have been enlarged to make them more visable.

**Figure 12:**
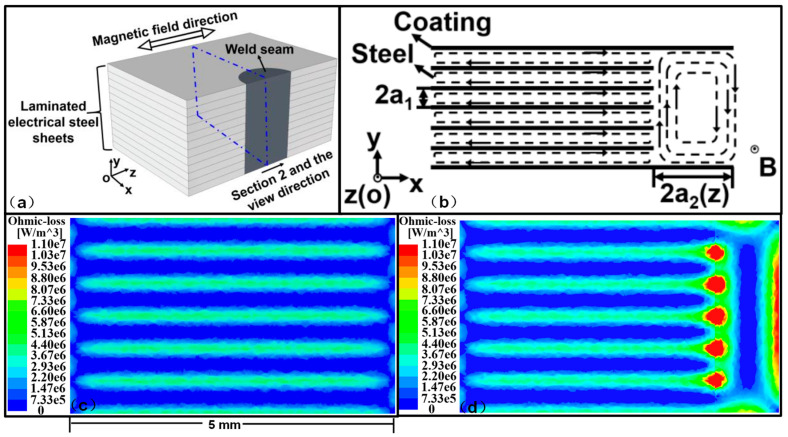
(**a**) Schematic of the welded electrical steel laminations; (**b**) schematic of the eddy current distribution at the cross section of the welded laminations under alternative magnetic field environment; (**c**) simulated eddy current loss distribution at the cross section of the laminations without welding; (**d**) simulated eddy current loss distribution at the cross section of the welded laminations through the weld seam [57].

The mechanical interlock and the fusion welding lead to the connection of the electrical steel laminations, which will increase the eddy current loss. Lamprecht et al. [62] developed a finite element model to identify the eddy current characteristics within the laminated stack and calculate the losses in the mechanically interlocked stack, the results showed that the eddy current losses increased for the interlocked sample in comparison to a perfectly insulated reference sample (Figure 11a,b). They also addressed the combination of the stacking impact and an additional electrical connection of the laminations as it may occur when the stator cores were pressed into an electrically conductive housing (Figure 11c,d). Wang et al. [57] developed a mathematical model based on the equivalent circuit method to calculate the eddy current loss in the welded electrical steel laminations, and the finite element model was also built to estimate the eddy current distribution in the local weld zone, as shown in Figure 12. The estimated eddy current loss by the mathematical model fit well with that by the finite element model, thus, the mathematical model could estimate the eddy current loss of the welded laminations in the actual motor with high calculation efficiency, while the finite element model could estimate the local distribution of the eddy current loss in the weld seam zone with high accuracy. Finally, both the mathematical model and the finite element model could behave as a useful tool to estimate the eddy current losses in the welded electrical steel laminations, towards high magnetic property welding of the laminations.

## 8. Stress Induced Magnetic Properties Degradation

**Figure 13:**
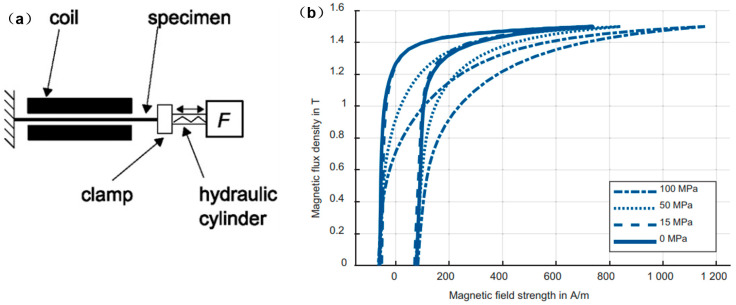
(**a**) Measurement principle for the load-related magnetic property; (**b**) measured hysteresis curves at different tensile stresses at 50 Hz for M400-50A [78]. In the experimental system, the force was loaded onto the specimens via the clamping jaws, where one side was fixed, and the other side was moveable and controlled by the pressure cylinder. The stress and magnetic flux were applied collinearly.

Manufacturing process, e.g., punching or cutting [63,64,65], welding [46], pressing and shrink-fitting [66] produced residual stress, which was also reported to lead to the degradation of the magnetic properties [67,68,69,70,71,72,73,74,75,76,77]. Karthaus et al. [78] developed an approach for modeling stress-dependent magnetic material properties such as magnetic flux density using a continuous local material model, and the presented model allowed a simple determination of model parameters by using stress-dependent magnetic material measurements, as shown in Figure 13a. The results of the mechanical stress-dependent hysteresis curves for tensile stress for 50 Hz were shown in Figure 13b. It can be observed that tensile stress caused a shear of the hysteresis curves. Thus, the magnetic properties such as magnetic remanence or iron losses were altered by the mechanical stress [78]. The following model could describe the magnetic flux density degradation because of the motivation of the mechanical stress [78], $B\left(\sigma ,H\right)=\mu_{0}H\left[\mu_{r}\left(\sigma =0,H\right)-\Delta \mu_{\sigma }\left(H\right)G\left(\sigma \right)\right]$, where H was the strength of the magnetic field, σ was the impressed mechanical stress,$\;\mu_{0}$ was the magnetic permeability of a vacuum, $\mu_{r}$ was the relative magnetic permeability of the material, $\Delta \mu_{\sigma }$ reflected the degradation of the magnetic permeability and G was the function describing the influence of the mechanical stress on the magnetizability of the material. Other papers also discussed the effects of the microstructure modified by the manufacturing process on the magnetic properties [79,80,81,82,83,84,85,86,87,88,89,90,91,92,93]. Generally, the hysteresis loss decreased and excess loss increased with increasing grain size [81,82]. In the future, it is important to correlate the magnetic properties with the microstructure of the material under different loads, e.g., grain size, grain orientation and magnetic domain. Besides, in-situ observation of the dynamic magnetic domain under the loading conditions will provide a better understanding of the magnetic properties degradation induced by the manufacturing process [94,95,96,97,98,99,100].

## 9. Comparison between Current Fusion Welding Methods

**Figure 14:**
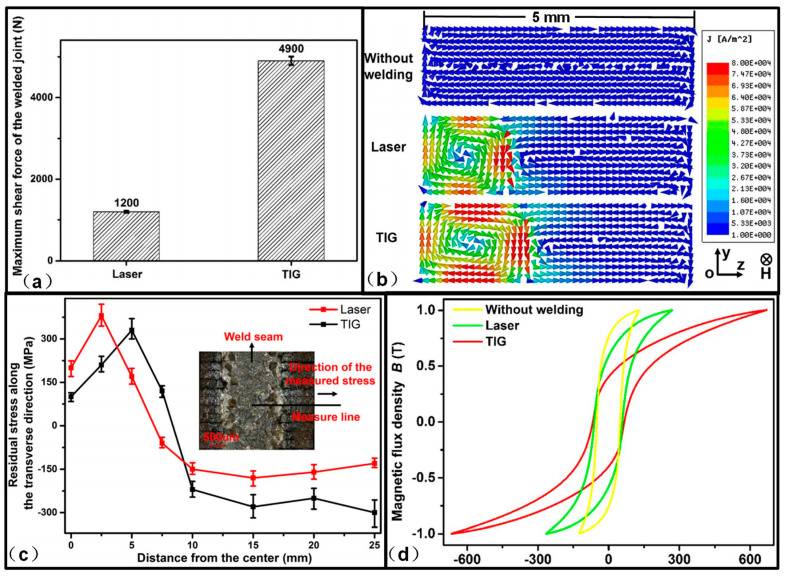
Comparison between TIG welding and laser welding: (**a**) Maximum shear strength; (**b**) eddy current distribution; (**c**) residual stress distribution; (**d**) hysteresis curve. The larger heat input in TIG welding leads to a larger cross section in the welded laminations, which then leads to larger shear strength and eddy current. The magnitude of the residual stress in TIG welding in the zone far away from the weld seam is larger than that in laser welding, and the hysteresis property in TIG welded sample has more severe degradation than that in laser-welded sample [51].

Compared to mechanical interlock and glue join, fusion welding is the most reliable method to join the electrical steel laminations for the high-performance motor application. Towards finding the best welding solution, various researchers have compared the current fusion welding methods [42,51,54,55]. Zhang et al. [51] compared the microstructure, mechanical performance, residual stress and magnetic properties of the electrical steel laminations welded by laser and TIG, as shown in Figure 14. Because of the larger heat input, the geometry size of the weld bead in TIG welding was larger than that in laser welding, which led to higher tensile shear strength. The eddy current loss in TIG-welded laminations was larger than that in laser-welded laminations because of the larger connection area between the laminations. Besides, the magnitude of the residual stress in TIG-welded laminations was also larger than that in laser-welded laminations, which led to severe degradation of the hysteresis property. To sum up, the heat input of laser was much more concentrated and controllable than that of TIG, and laser should be a better heat source for high quality welding of the electrical steel laminations.

**Figure 15:**
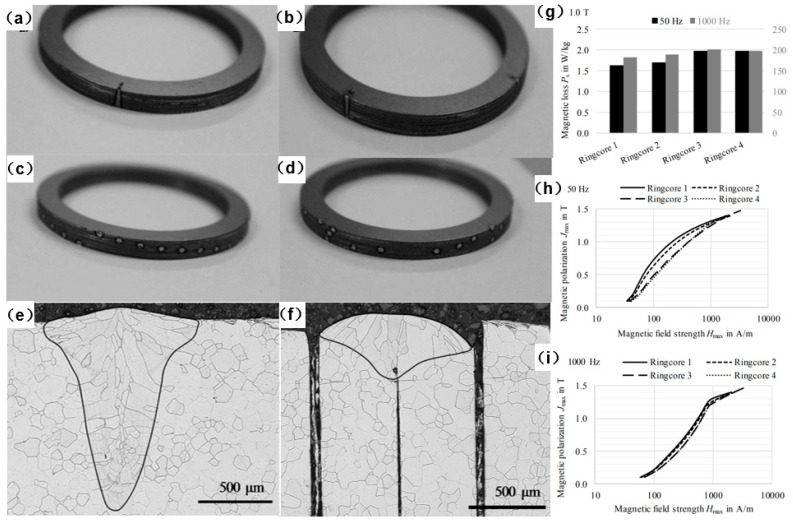
Comparison between line-welded laminations and spot-welded laminations: (**a**) Ring core 1, two linear welding lines; (**b**) ring core 2, four linear welding lines; (**c**) ring core 3, spirally oriented welding spots; (**d**) ring core 4, statistical distribution of welding spots; (**e**) cross section of the line weld; (**f**) cross section of the spot weld; (**g**) magnetic loss at 50 Hz and 1000 Hz and magnetic induction strength of 1.0 T for different cores. Magnetization curves of Ring cores 1–4 at different frequencies: (**h**) 50 Hz; (**i**) 1000 Hz [42]. The inner diameter of the Ring laminations was 48 mm and the outer diameter was 60 mm. As shown in (**h**,**i**), the magnetic polarization at a specific magnetic field strength follows the descending order of Ringcore 1, Ringcore 2, Ringcore 3 and Ringcore 4.

Leuning et al. [42] developed a novel welding strategy for electrical steel laminations using the statistical distribution of single laser welding spots alongside common linear welding lines across the entire height of the laminations, rather than the commonly used welding techniques of perpendicular lines that connect the whole laminations. The authors compared the novel welding strategy with the conventional ones, as shown in Figure 15. The experimental results showed that ring cores with perpendicular welding lines had a lower loss and better magnetization than both regarded modifications with distributed welding spots at low frequencies, which was due to the mechanical residual stress state of the ring cores induced by the thermal impact of welding. Additionally, the volume of the affected microstructure was smaller for the welding lines. At increasing frequencies, the eddy current component became dominant and the relative loss increase became distinctly smaller for the spot welded samples. The research result proved that the presented novel welding strategy was promising, especially as an approach for the high frequency applications.

**Figure 16:**
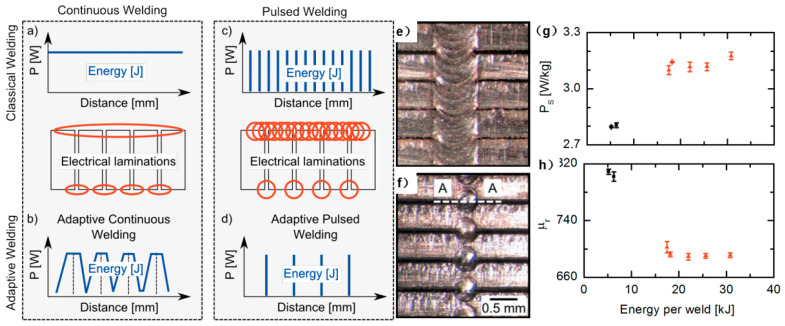
Comparison between line-welded laminations and adaptive pulsed-laser spot welded laminations: (**a**) Classic continuous welding; (**b**) adaptive continuous welding; (**c**) classic pulsed welding; (**d**) adaptive pulsed welding. A typical weld on laminations produced by: (**e**) classic pulsed-welding method; (**f**) adaptive pulsed-laser welding method. (**g**) the specific power losses (PS) and (**h**) the relative permeability (µr) as a function of the total-pulse energy for a real stack welded with the classic, continuous-welding technique (triangles) and adaptive pulsed method (squares) [55].

Vegelj et al. [55] developed and presented an experimental system for a new technique of adaptive and pulsed laser welding of electrical steel laminations, as shown in Figure 16. The system was based on on-line monitoring of the gap positions between the electrical laminations and thus enabled the precision welding at the interface of the electrical steel laminations. The experimental results showed that the developed pulsed spot welds produced lower specific power losses and increased the relative permeability of the samples in comparison with the conventional continuous laser welding that was widely used in industrial production. This method brought advantages to the production as well as to the final product and had great potential to be implemented in industry, though much work should be done for optimization. Ziegler et al. [101] concluded that quality control was also important for improving the welding quality, where process monitoring could be integrated into the welding system to control the quality and to optimize the parameters accordingly. The optical, spectral, thermal and acoustic sensors, which were increasingly being used separately or in combination, were particularly suitable for this purpose. For the evaluation of the measurement data, the intelligent approaches, e.g., machine learning, promised great potential. For the large-scale application of these novel welding technologies, it is important to have a comprehensive investigation about the welding quality, microstructure, mechanical properties and magnetic properties. Besides, the cost of both the welding system and welding each stator should also be considered, together with the robustness of the welding process and the life of the welding system.

## 10. Summary and Future Development

A comprehensive investigation about the effects of the joining process on the performance of the stator is required. The mechanical strength, fatigue life, and magnetic properties of the joined electrical steels are the indicators for the performance evaluation, especially the values of these indicators at an elevated temperature. Besides, the cost of each joining method also should be identified.

Much more work should be conducted to reveal the mechanism for magnetic properties degradation. The in-situ experiment may be a useful method to measure the magnetic properties of the joined electrical steels. For example, the temperature distribution in the joined electrical steels may be in-situ measured by the thermal imager [102], which could be used to validate the energy loss distribution in the electrical steels calculated by the thermal finite element model. The variation of the magnetic domain in the electrical steel at the external load condition under the alternating magnetic field environment may be in-situ measured by the neutron grating interferometry [103], magnetic force microscopy [104], magneto-optical indicator film [105], which could help understand the stress induced magnetic properties degradation.

Besides, there is still a huge space to optimize the process of joining the laminated electrical steels. More experiments could be conducted to build the relationship map between the process parameters and performance of the joined laminations, and the simulation model could help to understand the mechanism underlying the experimental phenomena.

In the current simulation model for welding of the laminated electrical steels, the birth and death element technique was adopted to describe the rapid increase of the thermal contact conductance when the interfaces between the laminations were melted due to laser irradiation. Though this method could characterize the effects of interfaces on the heat transfer during the welding process, the gap filling and residual stress evolution process still could not be analyzed. Finally, the thermal-mechanical-fluid coupled model will be developed to have an in-depth understanding of this welding process.

Most of the current research focused on evaluating the performance of the welded laminations, while did not reach the performance of the actual motor. The effects of the welding process on the performance of the motor investigated by both experiment and simulation are the future trend [38], which could build a direct relationship between the welding process and the final performance of the motor.

## Figures and Tables

**Table 1:** Representative research in the joining of laminated electrical steels.

Joining Method	Research Content	Year	Reference
Continuous laser welding	Strength: both the strength and the fatigue behavior of the weld material showed no appreciable difference to the base material; Microstructure: completely ferritic in both the base material and the weld seam; Defect: pores observed in the weld seam	2014	[37]
Continuous laser welding	Model for torsion strength: mathematical model with the function to estimate the strength of the welded laminations based on the welding parameters	2015	[56]
Continuous laser welding	Strength of the welded ring stator: increase with the heat input; Microstructure ferrite in the weld seam; Magnetic property: deteriorate with the heat input	2016	[40]
Continuous laser welding	Simulation of temperature distribution: discontinuous temperature distribution in the heat affected zone due to the hinder of the interface	2015	[47]
Continuous TIG welding	Strength, microstructure, magnetic property: TIG welded joint has higher strength, coarser grain and worse magnetic property than laser welded joint	2017	[51]
Continuous welding	Magnetic property: mathematical model and FEM model were developed to estimate the eddy current loss	2017	[57]
Mechanical joining	Interlaminar eddy currents mainly affect the iron loss of the local zone.	2017	[49]
Glue	Mechanical property: critical adhesive shear angle values of about 5° were obtained for all laminate samples, independent of the steel substrates used to create the laminates	2018	[26]
Adaptive pulsed spot welding	Possibility of the adaptive pulsed spot welding for laminated electrical steels was proved.	2014	[55]
Statistical distribution of single welding spots	The strategy of distributed welding spot shows promising results to decrease the magnetic deterioration, especially as an approach for higher frequency applications	2018	[42]

## Data Availability

The present manuscript is a review paper and does not contain original data. All data presented in the paper have been referenced appropriately.
